# A randomized, controlled trial evaluating the efficacy and safety of BTH1677 in combination with bevacizumab, carboplatin, and paclitaxel in first-line treatment of advanced non-small cell lung cancer

**DOI:** 10.1186/s40425-018-0324-z

**Published:** 2018-02-27

**Authors:** Walburga Engel-Riedel, Jamie Lowe, Paulette Mattson, J. Richard Trout, Richard D. Huhn, Michele Gargano, Myra L. Patchen, Richard Walsh, My My Trinh, Mariève Dupuis, Folker Schneller

**Affiliations:** 1 Kliniken der Stadt Köln gGmbH, Krankenhaus Merheim, Thoraxchirurgische u. Pneumologische Klinik, Ostmerheimer Str. 200, 51109 Köln, Germany; 2 Biothera Pharmaceuticals, Inc., 3388 Mike Collins Drive, Suite A, Eagan, MN 55121 USA; 3 Rutgers University, 82 Rittenhouse Circle, Newtown, PA 18940 USA; 4 Certara Strategic Consulting, 2000 Peel Street, Suite 570, Montréal, Québec, H3A2WS Canada; 5 Medical Clinic and Polyclinic of Klinikum rechts der Isar of Technical University Munich, Ismaninger Str. 22, 81675 Munich, Germany; 6 PresentAddress: Immuno Research, Inc., 3388 Mike Collins Drive, Suite B, Eagan, MN 55121 USA

**Keywords:** Immunotherapy, NSCLC, Bevacizumab, Beta-glucan

## Abstract

**Background:**

BTH1677, a beta-glucan pathogen-associated molecular pattern molecule, drives an anti-cancer immune response in combination with oncology antibody therapies. This phase II study explored the efficacy, pharmacokinetics (PK), and safety of BTH1677 combined with bevacizumab/carboplatin/paclitaxel in patients with untreated advanced non-small cell lung cancer (NSCLC).

**Methods:**

Patients were randomized to the BTH1677 arm (*N* = 61; intravenous [IV] BTH1677, 4 mg/kg, weekly; IV bevacizumab, 15 mg/kg, once each 3-week cycle [Q3W]; IV carboplatin, 6 mg/mL/min Calvert formula area-under-the-curve, Q3W; and IV paclitaxel, 200 mg/m^2^, Q3W) or Control arm (*N* = 31; bevacizumab/carboplatin/paclitaxel as above). Carboplatin/paclitaxel was discontinued after 4-6 cycles and patients who responded or remained stable received maintenance therapy with BTH1677/bevacizumab (BTH1677 arm) or bevacizumab (Control arm). Efficacy assessments, based on blinded central radiology review, included objective response rate (ORR; primary endpoint), disease control rate, duration of objective response, and progression-free survival. Overall survival and adverse events (AEs) were also assessed.

**Results:**

ORR was higher in the BTH1677 vs Control arm but the difference between groups was not statistically significant (60.4% vs 43.5%; *P* = .2096). All other clinical endpoints also favored the BTH1677 arm but none statistically differed between arms. PK was consistent with previous studies. Although a higher incidence of Grade 3/4 AEs occurred in the BTH1677 vs Control arm (93.2% vs 66.7%), no unexpected AEs were observed. Serious AEs and discontinuations due to AEs were lower in the BTH1677 vs Control arm.

**Conclusions:**

Improvements in tumor assessments and survival were observed with BTH1677/bevacizumab/carboplatin/paclitaxel compared with control treatment in patients with advanced NSCLC.

**Trial registration:**

ClinicalTrials.gov registration ID: NCT00874107. Registered 2 April 2009. First participant was enrolled on 29 September 2009.

## Background

Approximately 85% of all lung cancers are classified as non-small cell lung cancer (NSCLC) and the majority of patients present with locally advanced or metastatic disease [1]. Despite significant advances in NSCLC therapies, first-line treatment options for patients with advanced NSCLC still remain limited. Traditional platinum-based chemotherapy is still a mainstay therapy for most patients; however, this treatment approach generally provides only a short-lived benefit [2–4]. More recently, targeted therapies (eg, those targeting epidermal growth factor receptor [EGFR] gene mutations or anaplastic lymphoma kinase [ALK] translocations) have produced superior effects compared with chemotherapies for first-line management of advanced mutated NSCLC, but only small specific subtypes of NSCLC patients benefit from such treatments [2–4]. Several monoclonal antibody (MAb) therapies have also been approved for first-line treatment of advanced NSCLC, including bevacizumab (approved for non-squamous only), which targets vascular endothelial growth factor (VEGF) [5]; necitumumab (approved for squamous only) [6], which targets EGFR; and most recently, pembrolizumab, which targets the programmed death-1 (PD-1) immune checkpoint receptor on cytotoxic T cells [7]. Additional MAbs approved for second-line therapy, but possibly moving to front-line therapy, include ramucirumab [8], which targets VEGF receptor 2 (VEGFR2), as well as the anti-PD-1 and anti-programmed death ligand-1 (PD-L1) MAbs, nivolumab [9] and atezolizumab [10], respectively.

VEGF can compromise immune cell function in the tumor microenvironment, which may then become favorable for tumor survival and growth [11, 12]. Bevacizumab blocks VEGF receptor signaling [13]. Anti-VEGF agents can also normalize the tortuous vasculature of tumors and facilitate infiltration of lymphocytes [11]. Bevacizumab is approved by the Food and Drug Administration for unresectable, locally advanced, recurrent or metastatic non-squamous NSCLC in the first-line setting in combination with carboplatin/paclitaxel [14–16].

BTH1677 (Imprime PGG; β(1,6)-[poly-(1,3)-D-glucopyranosyl]-poly-β-(1,3)-D-glucopyranose; Biothera Pharmaceuticals, Inc., Eagan, MN) is a yeast-derived, water-soluble, 1,3-1,6 beta-glucan purified from the cell wall of a proprietary, non-recombinant, strain of *Saccharomyces cerevisiae.* It functions as a pathogen-associated molecular pattern (PAMP) molecule to support a coordinated innate and adaptive anti-cancer immune response in combination with oncology antibody therapies. When BTH1677 enters the blood, it is bound by endogenous plasma anti-beta-glucan antibodies (ABA) resulting in complement activation and opsonization with complement protein iC3b [17, 18]. The BTH1677/ABA/iC3b complex initially binds to innate immune effector cells through complement receptor 3 and Fc gamma receptor IIA (FcγIIA) [17, 18], activating innate immune cells and enabling direct killing of antibody-targeted tumor cells [17]. BTH1677 also enables remodeling of the tumor microenvironment, shifting the normally suppressive M2-state macrophages to a more M1 (pro-inflammatory) state [19–21], and promoting depletion and/or maturation of myeloid-derived suppressor cells in the tumor microenvironment [22, 23]. BTH1677 additionally activates antigen-presenting cells, driving co-stimulatory marker expression on macrophages and dendritic cells, as well as dendritic cell maturation, CD4 and CD8 T-cell expansion, and production of key anti-tumor cytokines (e.g., interferon gamma) [20, 24–27]. In murine syngeneic and xenogeneic tumor models, BTH1677 combined with various tumor-targeting MAbs [28–31], PD-1 and PD-L1 checkpoint-inhibiting MAbs [31–33], or VEGF/VEGFR2-targeted MAbs [22, 23, 31, 34–37] has resulted in greater suppression of tumor growth and longer survival than with either agent alone. In particular, 3 of these later studies have demonstrated synergy of BTH1677 when used in combination with bevacizumab in multiple lung cancer models [23, 35, 36]. Thus, the combination of BTH1677 and bevacizumab is a rational immunotherapy for treatment of cancer.

BTH1677 has been well tolerated after single doses up to 6 mg/kg and after 7 daily doses up to 4 mg/kg in healthy subjects. Pharmacokinetic (PK) parameters were proportional with dose [38]. Additionally, BTH1677 in combination with cetuximab, with or without irinotecan, was well tolerated with promising signs of efficacy in a phase Ib/II study in patients with recurrent or progressive metastatic colorectal cancer [39]. We also recently reported that BTH1677 combined with cetuximab/carboplatin/paclitaxel significantly improved ORR compared with cetuximab/carboplatin/paclitaxel in first-line treatment of patients with advanced NSCLC [40].

Here, we report results of a randomized, open-label, multicenter, phase II study evaluating the antitumor efficacy, safety, and PK profile of BTH1677 when combined with bevacizumab and concomitant carboplatin and paclitaxel therapy in patients with previously untreated, advanced NSCLC.

## Methods

### Study objectives

The primary objective was to evaluate the ORR (complete response [CR] + partial response [PR]) in each treatment arm. Secondary objectives included assessment of best response rate (CR, PR, or stable disease [SD] rates), disease control rate (DCR; CR + PR + SD), duration of objective tumor response (DOR), progression-free survival (PFS), and overall survival (OS) in each treatment arm. Safety within each arm and the PK profile of BTH1677 were also evaluated.

### Patient eligibility

Patients, 18 to 75 years of age, provided written informed consent, and had histologically or cytologically confirmed non-squamous stage IIIB or IV NSCLC according to American Joint Committee on Cancer Staging v6 [41]; measurable disease as defined by Response Evaluation Criteria in Solid Tumors (RECIST) v1.0; Eastern Cooperative Oncology Group performance status (ECOG PS) 0 or 1; life expectancy of > 3 months; adequate hematologic, renal, and hepatic function; and use of an effective contraceptive.

Exclusion criteria included prior systemic chemotherapy for lung cancer; previous radiation therapy to > 30% of active bone marrow or any radiation therapy within 3 weeks of Day 1; central nervous system metastases; uncontrolled hypertension; peripheral neuropathy ≥Grade 2; fever > 38.5 °C within 3 days of Day 1; active yeast infection; human immunodeficiency virus/acquired immune deficiency syndrome, hepatitis B, or hepatitis C; connective tissue or autoimmune disease; previous organ or progenitor/stem cell transplant; history of myocardial infarction or any other unstable, uncontrolled heart disease; second malignancy within the previous 5 years (other than basal cell carcinoma, cervical intra-epithelial neoplasm, or curatively treated prostate cancer); known hypersensitivity to baker’s yeast, murine proteins, or polyoxyethylated castor oil (Cremophor® EL); previous exposure to bevacizumab or BTH1677; or investigational therapy within 30 days prior to Day 1. Female patients were excluded if they were pregnant or breastfeeding.

### Study design and treatment plan

This randomized, open-label, multicenter, phase II study was performed at sites in Germany and the United States and was conducted in full accordance with the Good Clinical Practice: Consolidated Guideline approved by the International Conference on Harmonisation and all other applicable national and local laws/regulations. All study materials were approved by the governing ethics committee or institutional review board at each site.

The study was designed to test the null hypothesis that the true ORR was ≤30% vs the alternative hypothesis that the true ORR in the BTH1677 arm would be at least 50%. It was determined that 60 patients in the BTH1677 arm would provide 90% power for the hypotheses testing at an alpha level of 5%. With 2:1 randomization, a sample size of 30 patients was determined for the Control arm.

Patients in the BTH1677 and Control arms were dosed in 3-week cycles. On Days 1, 8, and 15 of each cycle, patients in the BTH1677 arm were administered 4 mg/kg of BTH1677 intravenously (IV) over 2 to 4 h (depending on patient weight and total dose administered). On Day 1 of each cycle, patients in each arm were administered 15 mg/kg of bevacizumab IV over 90 min. In the BTH1677 arm, bevacizumab was administered after BTH1677. On Day 2 of each cycle, IV carboplatin (dosed according to Calvert formula area under the curve [AUC] of 6 mg/mL•min over 30 min) and IV paclitaxel (200 mg/m^2^ over 3 h) were administered to all patients. No dosing occurred on Day 8 and Day 15 of the Control arm. Prior to each BTH1677 dosing, all patients were to receive low-dose corticosteroids and a histamine receptor-1 antagonist (e.g., 4 mg of dexamethasone orally and 50 mg of diphenhydramine IV). On Day 2 of each cycle, all patients were pre-medicated with the local clinic’s regimen of corticosteroids and antihistamines prior to carboplatin and paclitaxel therapy.

Carboplatin and paclitaxel administration continued for at least 4 cycles, but could continue for up to 6 cycles at the investigator’s discretion. Following completion of chemotherapy, patients who experienced a response (CR or PR) or had remained stable (SD) were eligible to continue on maintenance therapy receiving BTH1677/bevacizumab (BTH1677 arm) or bevacizumab (Control arm).

### Study assessments

Safety and tolerability were assessed by adverse events (AEs; National Cancer Institute Common Terminology Criteria for Adverse Events [CTCAE] v3.0), physical examinations, and laboratory tests.

Tumor response assessments were based on computed tomography (CT) scans performed every other cycle (i.e., at 6-week intervals). Blinded central radiology reviews were performed with tumor response assessed using a modified RECIST v1.0 criteria in which an initial response did not require a repeat assessment at a later time period for confirmation. All other RECIST v1.0 criteria were unmodified.

### Pharmacokinetics

Post-dosing blood samples were collected for PK assessments for BTH1677 on Day 1 of Cycles 1 and 3. Samples for BTH1677 trough level assessments were also obtained before weekly dosing on all other weeks of Cycles 1 and 2, as well as prior to dosing of Cycle 3, Day 1. Serum BTH1677 levels were measured by a beta-glucan specific enzyme-linked immunosorbent assay (ELISA) developed at Biothera Pharmaceuticals, Inc., which had a lower limit of detection of 1.2 ng/mL and a lower limit of quantitation of 4.7 ng/mL. Serum PK parameters were calculated using noncompartmental analysis (NCA) with NCA in WinNonlin® v5.2.

### Statistical analysis

**Fig. 1:**
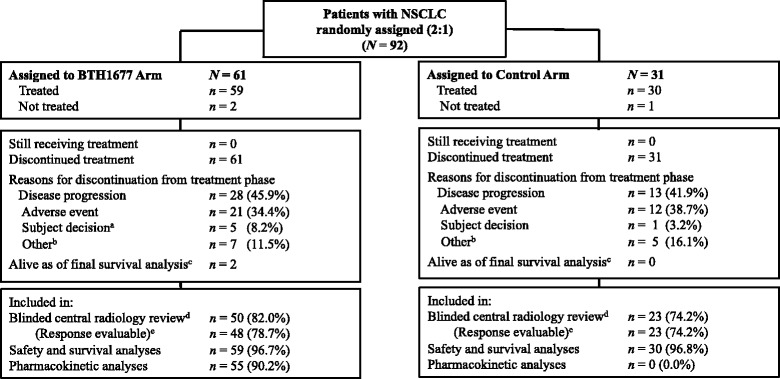
Patient disposition. ^a^ For 1 patient (*n* = 1), this occurred after primary data cut of 21 March 2014. ^b^ For the BTH1677 arm, ‘Other’ included investigator decision (*n* = 2), patient ineligibility (*n* = 2), radiotherapy following response (*n* = 1), tumor necrosis (*n* = 1), and sponsor ended study (*n* = 1); For the Control arm, ‘Other’ included planned surgery (*n* = 2), administrative decision (*n* = 1), investigator decision (*n* = 1), and sponsor ended study (*n* = 1). ^c^ Final data lock for OS analysis (16 March 2016) was performed approximately 3 years after the randomization date of last patient enrolled into the study. ^d^ Reasons for exclusion from efficacy analyses related to central radiology review in the BTH1677 arm were no evaluable baseline and/or post-baseline CT scan (*n* = 10; none of these patients had a best response of disease progression ie, clinical progression, reported by the investigator), and additionally 1 patient did not have histologically-confirmed NSCLC; in the Control arm, the primary reason for exclusion was no evaluable post-baseline CT scan (*n* = 8; 2 of these patients had a best response of disease progression ie, clinical progression, reported by the investigator). ^e^ Per protocol, to be “response evaluable” patients with best response of stable disease had to exhibit this response for at least 42 days (6 weeks) post randomization – if last scan occurred prior to this they were not “response evaluable”. Two patients in the BTH1677 arm were not evaluable for objective response, as their only on-study scan showed stable disease < 6 weeks post randomization. *Abbreviations: CT* computed tomography, *N* overall sample size, *n* number of patients, *NSCLC* non-small cell lung cancer, *OS* overall survival

The analysis populations for the blinded central radiology tumor assessments, safety and survival analyses, and PK analyses are shown by treatment arm in Fig. 1. The analysis population for tumor assessments (primary efficacy population) was comprised of all randomized patients who received any amount of bevacizumab, carboplatin, or paclitaxel, with or without BTH1677, and who had an evaluable baseline CT scan assessment and at least 1 evaluable post-baseline CT scan assessment. The safety and survival populations were comprised of all randomized patients who received any amount of study drugs. The PK population was comprised of all patients who had at least 75% of the PK measurements available for any particular treatment cycle data set. The primary analysis data lock (which included all analyses except final OS) occurred on 21 March 2014. Data lock for final survival analysis occurred on 16 March 2016, which was approximately 3 years after the randomization date of the last patient enrolled into the study.

Efficacy and safety measures are displayed by treatment arm. Categorical data are presented by *n* and % for each category and continuous data are presented by mean and standard deviation. Kaplan-Meier estimates were utilized for time-to-event analyses and, where appropriate, 95% confidence intervals (CI) are provided. Comparisons between treatment arms were performed at a 0.05 level of significance. AEs are summarized by system organ class using the Medical Dictionary for Regulatory Activities v15.0.

## Results

### Patient disposition

Between 29 September 2009 and 08 March 2013, a total of 92 patients with NSCLC were randomly assigned 2:1 to the BTH1677 or the Control arm. As shown in Fig. 1, 59 patients in the BTH1677 arm and 30 patients in the Control arm were treated and included in the safety and survival analyses. The blinded central radiology review primary efficacy population included 50 patients (48 patients response evaluable) in the BTH1677 arm and 23 patients in the Control arm. The primary reason for patient exclusion from the primary efficacy population was that an evaluable baseline or post-baseline CT scan was not available or acceptable to the central radiology reviewers. The primary reason for treatment discontinuation in each arm was disease progression (BTH1677, 45.9%; Control, 41.9%).

Patient demographics and disease characteristics at baseline are shown in Table 1. The BTH1677 and Control arms were similar with respect to age, sex, race, as well as time from initial tumor diagnosis to diagnosis of stage IIIB/IV NSCLC and diagnosis of stage IIIB/IV NSCLC to randomization. The percentage of patients with ECOG PS status of 1 vs 0 was higher in the BTH1677 arm (47.5%) than in the Control arm (33.3%). The percentage of patients who received prior cancer treatments (radiotherapy, surgery, chemotherapy, hormonal, or other) was also higher in the BTH1677 arm compared with the Control arm.

**Table 1:** Patient demographics and disease characteristics at baseline (Safety population)

	BTH1677/ Bevacizumab/Carboplatin/ Paclitaxel (*N* = 59)	Bevacizumab/ Carboplatin/ Paclitaxel (*N* = 30)
Age (years)		
Median (range)^a^	59 (43, 76)	58 (28, 75)
Sex, n (%)		
Male	26 (44.1)	14 (46.7)
Female	33 (55.9)	16 (53.3)
Race, n (%)		
White	57 (96.6)	30 (100.0)
Black	1 (1.7)	0
Asian or Pacific Islander	1 (1.7)	0
ECOG Performance Status, n (%)		
0	31 (52.5)	20 (66.7)
1	28 (47.5)	10 (33.3)
Disease stage at randomization		
Stage IIIB	0	0
Stage IV	59 (100.0)	30 (100.0)
Time from initial tumor diagnosis to diagnosis of stage IIIB/IV NSCLC (days)^b^		
Median (range)	0 (0, 3158)	0 (0, 0)
Time from diagnosis of stage IIIB/IV NSCLC to randomization (days)^c^		
Median (range)	18 (1, 168)	17.5 (7, 77)
Time from initial tumor diagnosis to randomization (days)^d^		
Median (range)	20 (1, 3171)	17.5 (7, 77)
Prior cancer treatment, n (%)		
Radiotherapy	2 (3.4)	0
Surgery	8 (13.6)	2 (6.7)
Chemotherapy, hormonal, or other^e^	2 (3.4)	0

*Abbreviations: ECOG* Eastern Cooperative Oncology Group, *N* overall sample size, *n* number of patients, *NSCLC* non-small cell lung cancer

^a^Inclusion criteria restricted patients to 18 to 75 years of age but one 76-year-old patient was inadvertently enrolled in the study

^b^Time from initial tumor diagnosis to diagnosis of Stage IIIB/IV NSCLC = Stage IIIB/IV diagnosis date - initial tumor diagnosis date

^c^Time from diagnosis of Stage IIIB/IV NSCLC to randomization = Date of randomization - Stage IIIB/IV diagnosis date

^d^Time from initial tumor diagnosis to randomization = Date of randomization –initial tumor diagnosis date

^e^Two patients in the BTH1677 arm received prior chemotherapy for lung cancer. Both patients were previous responders and received the prior chemotherapy greater than 6 months prior to enrollment

### Efficacy

#### Tumor-associated assessments

**Fig. 2:**
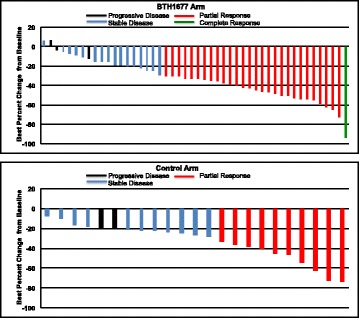
Maximum reduction from baseline in sum of longest diameters (SLD) for target lesions. Blinded central radiology review data from individual patients in the primary efficacy population

**Fig. 3:**
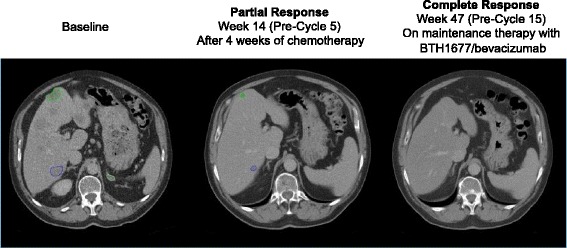
CT scans showing continued reduction in tumor burden while on BTH1677 maintenance therapy. Target lesion locations at baseline included left hilum, mediastinal lymph nodes, adrenals, and liver. The patient remained on study and in CR at the time of the primary analysis (19 weeks later). *Abbreviations: CR* complete response, *CT* computed tomography

Tumor-associated assessments were based on the blinded central radiology review of the primary efficacy population. Although the between-group difference was not statistically significant, compared with the Control arm, patients in the BTH1677 arm had a higher ORR (60.4% vs 43.5%; *P* = .2096) (Table 2). One patient in the BTH1677 arm had a CR while no patients in the Control arm had CR (Table 2 and Fig. 2). While on maintenance therapy, continued reduction in the sum of the longest diameters of target lesions of > 20 mm and > 10 mm, respectively, occurred in 7% and 20% of the BTH1677 arm patients, but in none of the Control arm patients (Table 3). One of these continued reductions included a BTH1677 arm patient who had a CR at treatment week 47 (Fig. 3); this patient remained in the study with a CR for an additional 19 weeks until the study was closed for primary analysis.

**Table 2:** Tumor-associated assessments based on a blinded central radiology review of the primary efficacy population

	BTH1677/Bevacizumab/ Carboplatin/Paclitaxel (*N* = 48)		Bevacizumab/ Carboplatin/Paclitaxel (*N* = 23)		
	n (%)	95% CI	n (%)	95% CI	*P* value^f^
Objective Response Rate^a,b^	29 (60.4)	(45.3, 74.2)	10 (43.5)	(23.2, 65.5)	0.2096
Disease Control Rate^b,c^	45 (93.8)	(82.8, 98.7)	21 (91.3)	(72.0, 98.9)	0.6563
Best Observed Response^b^					
Complete response	1 (2.1)	(0.1, 11.1)	0	NA	
Partial response	28 (58.3)	(43.2, 72.4)	10 (43.5)	(23.2, 65.5)	0.3113
Stable disease	16 (33.3)	(20.4, 48.4)	11 (47.8)	(26.8, 69.4)	0.2992
Progressive disease	3 (6.3)	(1.3, 17.2)	2 (8.7)	(1.1, 28.0)	0.6563
Duration of Objective Tumor Response^d^					HR (95% CI)^g^
Patients with objective response (CR + PR)	29		10		
Patients (% responding patients) with known duration (uncensored)	9 (31.0)		3 (30.0)		
Patients (% responding patients) with unknown duration (censored)	20 (69.0)		7 (70.0)		
Duration of objective response (months)					
Median (95% CI)	10.3 (5.6, NE)		5.6 (1.5, NE)		0.92 (0.27, 4.20)
Log-rank *P* value					0.9040
Progression-Free Survival^e^					HR (95% CI)^g^
Patients with PD or died, n (%)	17 (34.0)		6 (26.1)		
Patients censored, n (%)	33 (66.0)		17 (73.9)		
Progression-free survival (months)					
Median (95% CI)	11.6 (7.0, NE)		9.6 (7.1, NE)		1.31 (0.54, 3.65)
Log-rank *P* value					0.5639

*Abbreviations: CI* confidence interval, *CR* complete response, *NA* not applicable, *PD* progressive disease, *PR* partial response, *HR* hazard ratio, *n* number of patients, *N* overall sample size, *NE* not estimable

^a^Objective response rate was defined as the percent of central radiology review response-evaluable patients experiencing a best overall tumor

response of either CR or PR at any time on study according to modified RECIST v1.0 criteria

^b^Tumor response data reported as the number (n) and percent (%) of response-evaluable patients and the 95% exact binomial confidence interval

^c^Disease control rate was defined as the percent of blinded central radiology review response-evaluable patients experiencing a best overall tumor response of CR, PR, or stable disease (SD) per modified RECIST v1.0 criteria

^d^Duration of objective response (months) was based on Kaplan-Meier estimates and was measured from the time at which criteria were met for CR or PR (whichever status was recorded first) until the first date on which recurrence or progressive disease (PD) was objectively documented per modified RECIST v1.0. Patients who did not progress as of the data cutoff date were censored at their last tumor assessment

^e^Progression-free survival (PFS) (months) was based on Kaplan-Meier estimates from the total blinded central radiological review population (BTH1677/Bevacizumab/Carboplatin/Paclitaxel, *n* = 50 and Bevacizumab/Carboplatin/Paclitaxel, n = 23) and was defined as the time from randomization to the first date of documented PD or death. PD was identified by radiologic PD according to modified RECIST v1.0. If a patient received any further anticancer therapy without prior documentation of PD, the patient was censored at the date of last imaging assessment before the treatment. Patients who were lost to follow-up or who were alive without PD as of the data cut-off date were censored at the last imaging assessment date or at the analysis data cut-off date, whichever came first

^f^*P* value was obtained using Fisher’s Exact Test

^g^Hazard ratio (95% exact binomial CI) was based on Cox proportional hazards model with treatment as factor

**Table 3:** Change in target lesion sum of longest diameters from post-chemotherapy baseline to post-chemotherapy nadir

Length of SLD Reduction (mm)	BTH1677/ Bevacizumab/Carboplatin/ Paclitaxel^a^ (*N* = 30)	Bevacizumab/Carboplatin/ Paclitaxel^a^ (*N* = 13)
> 20	2 (7%)	0
> 10	6 (20%)	0
> 5	10 (33%)	2 (15%)

*Abbreviations: N* overall sample size, *SLD* sum of longest diameters

^a^Number of patients continuing in study on maintenance therapy with at least 2 post-chemotherapy tumor assessments based on blinded central radiology review of the primary efficacy population

**Fig. 4:**
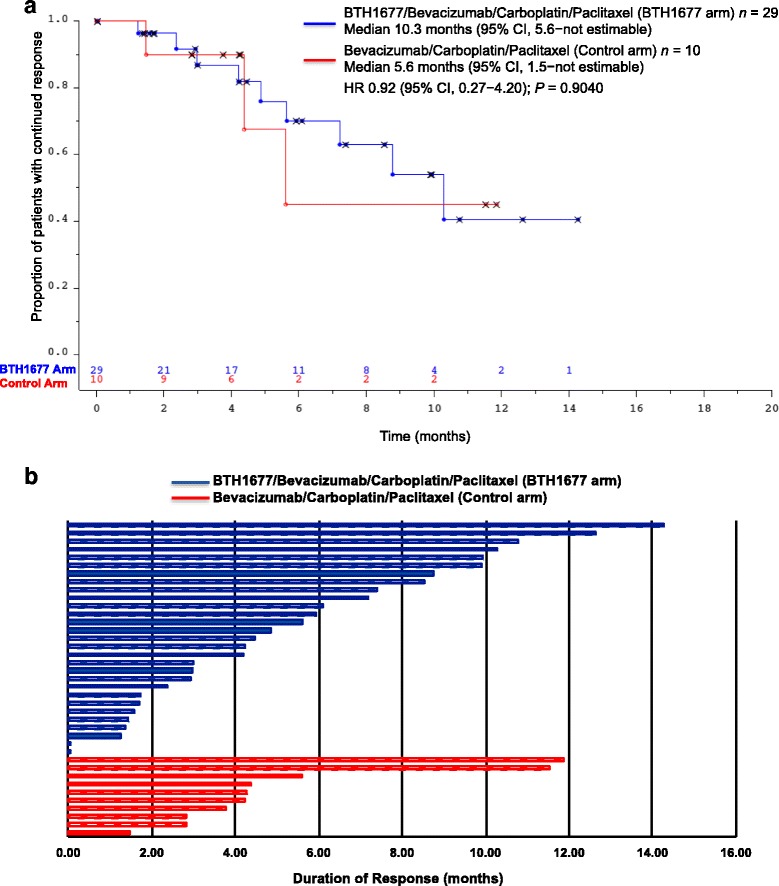
Duration of objective response. **a** The Kaplan-Meier duration of response (months) curve from patients in the primary efficacy population based on blinded central radiology review. *Abbreviations: CI* confidence interval, *n* number of patients, *HR* hazard ratio. **b** Plot of duration of response for primary efficacy population by patient based on blinded central radiology review. Hatched blue and red bars indicate censoring in the BTH1677 and Control arms, respectively

Of the patients with an objective response (BTH1677 *n* = 29; Control *n* = 10), the median DOR was 10.3 months in the BTH1677 arm (95% CI: 5.6, not estimable) vs 5.6 months (95% CI: 1.5, not estimable) in the Control arm (HR 0.92 [95% CI: 0.27, 4.20]; *P* = .9040; Table 2 and Fig. 4a). DOR by patient is illustrated in Fig. 4b. Of the patients with progressive disease or death (BTH1677 *n* = 17; Control *n* = 6), the PFS was 11.6 months in the BTH1677 arm and 9.6 months in the Control arm (HR 1.31 [95% CI: 0.54, 3.65]; *P* = .5639; Table 2).

#### Overall survival

**Fig. 5:**
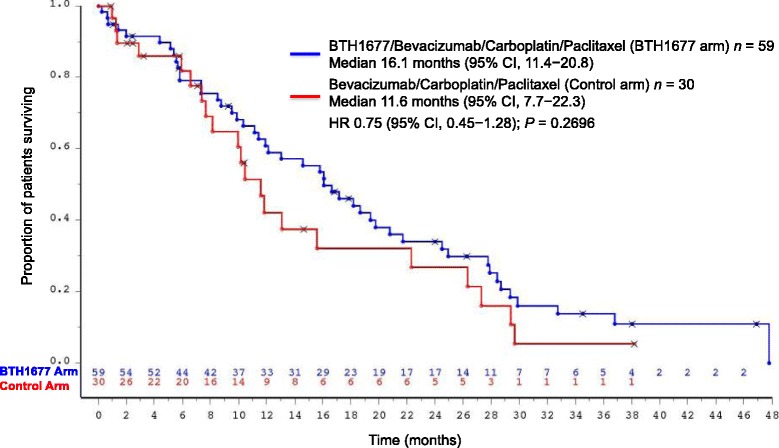
Overall survival. The Kaplan-Meier overall survival (months) curve from patients in the safety population. X indicates censored patients. *Abbreviations: CI* confidence interval, *n* number of patients, *HR* hazard ratio

The OS Kaplan-Meier curves for the BTH1677 and Control arms are shown in Fig. 5. The median OS of patients in the BTH1677 arm was 16.1 months (95% CI: 11.4, 20.8) compared with 11.6 months (95% CI: 7.7, 22.3) in the Control arm. However, the difference in median OS was not statistically significant between arms (HR 0.75 [95% CI: 0.45, 1.28]; *P* = .2696).

### Safety

All patients receiving any treatment (BTH1677 *n* = 59; Control *n* = 30) were included in the safety population, and the majority of these patients (≥98%) experienced at least 1 AE. Grade 3 or Grade 4 AEs occurred at a higher incidence among patients in the BTH1677 arm compared with patients in the Control arm (93.2% vs 66.7%, respectively). However, the incidence of serious AEs (40.7% vs 43.3%) and AEs leading to discontinuation (35.6% vs 40%) was slightly lower in the BTH1677 arm than the Control arm, respectively. In the BTH1677 arm, 22.0% and 28.8% of the AEs were considered probably and possibly related to BTH1677, respectively. These data are summarized in Table 4.

**Table 4:** Overview of safety outcomes (Safety population)

Adverse events (AEs), n (%)	BTH1677/ Bevacizumab/ Carboplatin/Paclitaxel (*N* = 59)	Bevacizumab/ Carboplatin/ Paclitaxel (*N* = 30)
Any AE	58 (98.3)	30 (100.0)
NCI/CTCAE Grade 3 or 4 AEs	55 (93.2)	20 (66.7)
Serious AEs	24 (40.7)	13 (43.3)
BTH1677-related AEs		
Probably related	13 (22.0)	NA
Possibly related	17 (28.8)	NA
AEs leading to discontinuation	21 (35.6)	12 (40.0)

*Abbreviations: AE* adverse events, *N* overall sample size, *n* number of patients, *NA* not applicable, *NCI/CTCAE* National Cancer Institute Common Terminology Criteria for Adverse Events

AEs of any grade that occurred in ≥10% of patients in the BTH1677 and Control arms are presented in Table 5. Gastrointestinal, general, hematological, and skin disorders were commonly reported AEs among both arms. Of the AEs reported in these categories, neutropenia, leukopenia, nausea, constipation, vomiting, abdominal pain upper, stomatitis, pyrexia, and chills occurred at an incidence at least 5% higher in the BTH1677 arm than in the Control arm; of these AEs, stomatitis and chills occurred exclusively in the BTH1677 arm (10.2% vs 0% and 18.6% vs 0%, respectively). In contrast, diarrhea and chest pain occurred at an incidence at least 5% higher in the Control arm than in the BTH1677 arm.

**Table 5:** Any grade AEs (≥10%) and corresponding Grade 3 or 4 AEs (Safety population)

	BTH1677/ Bevacizumab/ Carboplatin/Paclitaxel (*N* = 59)		Bevacizumab/ Carboplatin/Paclitaxel (*N* = 30)	
Adverse events (AEs), n (%)	All AEs	Grade 3 or Grade 4 AEs	All AEs	Grade 3 or Grade 4 AEs
Patients with at least 1 AE	58 (98.3)	55 (93.2)	30 (100.0)	20 (66.7)
Blood and lymphatic system disorders				
Neutropenia	26 (44.1)	23 (39.0)	10 (33.3)	8 (26.7)
Thrombocytopenia	20 (33.9)	11 (18.6)	10 (33.3)	5 (16.7)
Anemia	14 (23.7)	2 (3.4)	8 (26.7)	1 (3.3)
Leukopenia	9 (15.3)	3 (5.1)	2 (6.7)	1 (3.3)
Gastrointestinal disorders				
Nausea	40 (67.8)	5 (8.5)	16 (53.3)	0
Constipation	25 (42.4)	0	9 (30.0)	0
Vomiting	19 (32.2)	2 (3.4)	7 (23.3)	0
Diarrhea	12 (20.3)	2 (3.4)	8 (26.7)	1 (3.3)
Abdominal pain upper	6 (10.2)	1 (1.7)	1 (3.3)	0
Stomatitis	6 (10.2)	1 (1.7)	0	0
General disorders and administration-site conditions				
Fatigue	33 (55.9)	1 (1.7)	18 (60.0)	1 (3.3)
Pyrexia	12 (20.3)	2 (3.4)	3 (10.0)	0
Chills	11 (18.6)	1 (1.7)	0	0
Mucosal inflammation	6 (10.2)	0	3 (10.0)	0
Chest pain	3 (5.1)	0	5 (16.7)	0
Infections and infestations				
Nasopharyngitis	6 (10.2)	0	3 (10.0)	0
Urinary tract infection	2 (3.4)	1 (1.7)	3 (10.0)	0
Injury, poisoning, and procedural complications				
Infusion-related reaction	7 (11.9)	1 (1.7)	2 (6.7)	1 (3.3)
Investigations				
Hemoglobin decreased	4 (6.8)	1 (1.7)	3 (10.0)	2 (6.7)
Platelet count decreased	3 (5.1)	2 (3.4)	3 (10.0)	1 (3.3)
Metabolism and nutritional disorders				
Decreased appetite	17 (28.8)	1 (1.7)	11 (36.7)	0
Hypokalemia	6 (10.2)	1 (1.7)	0	0
Musculoskeletal and connective tissue disorders				
Arthralgia	19 (32.2)	1 (1.7)	6 (20.0)	0
Pain in extremity	15 (25.4)	0	3 (10.0)	0
Back pain	11 (18.6)	1 (1.7)	4 (13.3)	0
Myalgia	12 (20.3)	1 (1.7)	2 (6.7)	0
Nervous system disorders				
Polyneuropathy	16 (27.1)	3 (5.1)	6 (20.0)	0
Headache	9 (15.3)	1 (1.7)	4 (13.3)	0
Neuropathy peripheral	6 (10.2)	0	5 (16.7)	0
Dizziness	7 (11.9)	0	3 (10.0)	0
Paresthesia	7 (11.9)	0	2 (6.7)	0
Dysgeusia	6 (10.2)	0	2 (6.7)	0
Psychiatric disorders				
Insomnia	9 (15.3)	0	2 (6.7)	0
Sleep disorder	5 (8.5)	0	4 (13.3)	0
Respiratory, thoracic and mediastinal disorders				
Dyspnea	18 (30.5)	3 (5.1)	8 (26.7)	0
Cough	18 (30.5)	2 (3.4)	7 (23.3)	0
Epistaxis	14 (23.7)	0	10 (33.3)	0
Oropharyngeal pain	8 (13.6)	0	2 (6.7)	0
Skin and subcutaneous tissue disorders				
Alopecia	28 (47.5)	2 (3.4)	14 (46.7)	0
Vascular disorders				
Hypertension	14 (23.7)	3 (5.1)	7 (23.3)	1 (3.3)

*Abbreviations: AE* adverse events, *N* overall sample size, *n* number of patients

For AEs of any grade that occurred in ≥10% of patients, those that were Grade 3 or Grade 4 are also presented in Table 5. For purposes of comparison here, Grade 3 or Grade 4 AEs that occurred in ≥5% of patients in either treatment arm included neutropenia (39.0% BTH1677 vs 26.7% Control), thrombocytopenia (18.6% BTH1677 vs 16.7% Control), leukopenia (5.1% BTH1677 vs 3.3% Control), nausea (8.5% BTH1677 vs 0% Control), hemoglobin decreased (1.7% BTH1677 vs 6.7% Control), polyneuropathy (5.1% BTH1677 vs 0% Control), dyspnea (5.1% BTH1677 vs 0% Control), and hypertension (5.1% BTH1677 vs 3.3% Control).

Eight deaths (7 in the BTH1677 arm; 1 in the Control arm) were reported in the treatment phase of within 30 days of the last dose of study medication. Six of the 7 deaths in the BTH1677 arm were due to disease progression or complications of disease progression (e.g., pneumonia due to lung cancer, pneumothorax); the other death was due to intracranial hemorrhage that occurred subsequent to administration of anticoagulant for a blood clot in the lung. The 1 death in the Control arm was due to complications of disease progression (pneumonia due to lung cancer).

### BTH1677 pharmacokinetics

BTH1677 serum concentrations in both cycles were above the limit of quantitation (4.7 ng/mL) for all but 1 patient. Individual t_max_ values generally coincided with the end of infusion or shortly thereafter. Mean serum trough levels of BTH1677 appeared to plateau as of Day 15 of Cycle 1, suggesting that steady state was achieved as of Day 15. Table 6 summarizes the PK parameters of BTH1677 from Cycle 1/Day 1 (*n* = 53) and Cycle 3/Day 1 (*n* = 42). Geometric mean C_max_ of serum BTH1677 was similar in Cycle 1 (49.77 μg/mL) and Cycle 3 (60.50 μg/mL). AUC_0-24_ of BTH1677 was also similar in Cycle 1 (399.8 μg•hr./mL) and Cycle 3 (464.4 μg•hr./mL), with little to no accumulation of BTH1677 being observed. No notable difference in other PK parameters (CL, t_max_, and V_ss_) between Cycle 1 and Cycle 3 were observed. A longer elimination half-life (t_1/2_) was observed in Cycle 1 (17.56 h) than Cycle 3 (7.17 h); however, these results should be interpreted with caution given the difference in sampling periods between Cycle 1 (up to 168 h post dosing) and Cycle 3 (only up to 24 h post dosing).

**Table 6:** Summary of BTH1677 pharmacokinetics parameters

Parameters	Geometric Mean (CV%)	
	BTH1677/Bevacizumab/ Carboplatin/Paclitaxel	
	Cycle 1/Day 1	Cycle 3/Day 1
N	53^b^	42
AUC_0–last_ (μg•hr./mL)	614.6 (52.0)	423.5 (48.1)
AUC_0–24_ (μg•hr./mL)	399.8 (37.3)	464.4 (38.9)^d^
AUC_0–∞_ (μg•hr./mL)	635.9 (49.5)^c^	518.6 (42.2)^e^
C_max_ (μg/mL)	49.77 (36.1)	60.50 (49.2)
CL (L/h)	0.441 (47.3)^c^	0.548 (41.6)^e^
t_1/2_ (hr)	17.56 (36.9)^c^	7.17 (35.3)^e^
t_max_ (hr)^a^	2.48 (1.52, 5.90)	2.37 (1.92, 6.88)
V_ss_ (L)	5.37 (42.4)^c^	4.13 (51.5)^e^
R (AUC)	NA	1.10 (27.3)^d^

*Abbreviations: AUC*_*(0-last)*_ area under the serum concentration-time curve from time 0 to the time of the last measurable concentration, *AUC*_*0–24*_ area under the serum concentration-time curve from time 0 to 24 h, *AUC*_*0–∞*_ area under the serum concentration-time curve from time 0 to infinity, *C*_*max*_ maximum serum concentration, *CL* systemic clearance, *CV* coefficient of variation, *hr.* hour, *L* liter, *mL* milliliter, *N* overall sample size, *NA* not applicable, *R (AUC)* accumulation ratio calculated as AUC_0-24_ (Cycle 3)/AUC_0-24_ (Cycle 1), *t*_*1/2*_ elimination half-life, *t*_*max*_ time of maximum concentration, μg microgram, *V*_*ss*_ volume of distribution at steady-state

^a^Median (range)

^b^Although 2 other patients met the basic criteria for inclusion in the PK population (ie, having at least 75% of the PK measurements available for any particular treatment cycle data set), 2 patients were subsequently excluded from analysis. One patient received a second unplanned BTH1677 infusion on Day 2 of Cycle 1 and a second patient had a longer infusion time (5.58 h) relative to the other patients for Cycle 1/Day 1. Both of these differences were deemed to likely result in PK differences relative to other patients in Cycle 1 and were excluded

^c^N = 50

^d^*N* = 37

^e^N = 31

## Discussion

It has only been in recent years that harnessing the immune system in the fight against cancer has become well appreciated, especially with respect to therapy of NSCLC [42–46]. BTH1677 is a novel PAMP molecule capable of inducing coordinated innate and adaptive immune responses. BTH1677 is being developed for the treatment of cancer in combination with tumor-targeted, anti-angiogenic, and checkpoint inhibitor antibodies. Here we report the effects of BTH1677 combined with the anti-angiogenic MAb, bevacizumab, and platinum-based chemotherapy in first-line treatment of advanced non-squamous NSCLC patients.

Previously the ECOG and AVAiL trials [14–16] evaluated clinical outcomes when bevacizumab was added to a platinum-based chemotherapy backbone in patients with advanced non-squamous NSCLC in a first-line setting. In particular, the ECOG study evaluated the same chemotherapy backbone of carboplatin and paclitaxel as our current study [14]. In this randomized, open-label, phase II study, ORR, DOR, PFS, and OS were higher in patients receiving BTH1677 in combination with bevacizumab, carboplatin, and paclitaxel compared with patients receiving bevacizumab, carboplatin, and paclitaxel alone. As statistical significance was not achieved in any of the assessments, all results should be interpreted carefully. As a phase II study, its main goal was to better define the point estimates and variability of the efficacy parameters to be used for statistical design in a later registrational trial. The consistent positive trend across multiple clinical endpoints suggests a benefit with the addition of BTH1677 to bevacizumab, carboplatin, and paclitaxel and is consistent with synergistic effects seen with preclinical in vivo studies with BTH1677 added to bevacizumab therapy in murine lung cancer models [23, 35, 36]. It should also be noted that the Control arm in this trial performed similarly to, or better than, the bevacizumab/carboplatin/paclitaxel arm in the ECOG 4599 study (ECOG ORR 35% vs our 43.5%; ECOG PFS 6.2 months vs our 9.6 months; ECOG OS 12.3 months vs our 11.6 months), giving credibility to the further improvements of 60.4%, 11.6 months, and 16.1 months observed for these respective endpoints with the addition of BTH1677.

The most frequently reported AE system organ classes were blood and lymphatic system disorders, gastrointestinal disorders, and general disorders/administration site conditions, which are consistent with AE classes typically associated with the underlying disease and/or the backbone therapy. Although an overall higher incidence of Grade 3 or Grade 4 AEs was reported in the BTH1677 arm than the Control arm, fewer patients in the BTH1677 arm than in the Control arm discontinued therapy due to AEs. It has subsequently been observed in ongoing cancer trials that infusion reactions may be associated with BTH1677 administration in some patients and may consist of various constellations of transient fever/chills, headache, dyspnea/cough, nausea, vomiting, abdominal pain, back pain, and myalgia/arthralgia; such events were observed at Grade 3 levels and contributed to the overall higher incidence of Grade 3 or Grade 4 AEs in the BTH1677 arm of this trial. Fatal AEs reported in the BTH1677 arm were generally associated with progression of disease. Overall, these safety findings further support a safety profile for BTH1677, which is consistent with our previous reports with BTH1677 alone [38] or in combination with MAb and chemotherapy in patients with metastatic colorectal cancer [39] and advanced NSCLC [40].

Currently, the most notable immunotherapies recognized as impacting clinical outcomes in oncology are the checkpoint inhibitors (CPIs), among which pembrolizumab [7], nivolumab [9], and atezolizumab [10] (PD-1/PD-L1 CPIs) have been approved for various stages of NSCLC. In early-phase studies, these agents are also being evaluated in combination with anti-angiogenic agents, including bevacizumab (with and without concomitant chemotherapy), with results pending [47]. These studies further support the concept of our study design to combine immunotherapy and an anti-angiogenic agent in hope of identifying an improved therapeutic approach for NSCLC patients.

## Conclusion

In conclusion, BTH1677 in combination with bevacizumab/carboplatin/paclitaxel as a first-line treatment of advanced NSCLC led to improvements in ORR, DOR, PFS, and OS compared with the Control arm. No major safety concerns were noted with the BTH1677 combination therapy. Ongoing studies are continuing to explore this novel PAMP immune modulator as an adjunct to antibody-based therapy, including checkpoint inhibitor therapies, for patients with NSCLC (NCT03003468) as well as other cancers (NCT02086175; NCT02981303).

## Abbreviations

ABA: Anti-beta-glucan antibodies
AE: Adverse events
ALK: Anaplastic lymphoma kinase
AUC: Area under the curve
AUC_0–24_: Area under the serum concentration-time curve from time 0 to 24 h
CI: Confidence intervals
CL: Systemic clearance
C_max_: Maximum serum concentration
CPI: Checkpoint inhibitors
CR: Complete response
CT: Computed tomography
CTCAE: Common Terminology Criteria for Adverse Events
DOR: Duration of objective tumor response
ECOG: Eastern Cooperative Oncology Group
EGFR: Epidermal growth factor receptor
ELISA: Enzyme-linked immunosorbent assay
HR: Hazard ratio
IV: Intravenously
MAb: Monoclonal antibody
NCA: Noncompartmental analysis
NSCLC: Non-small cell lung cancer
ORR: Objective response rate
OS: Overall survival
PAMP: Pathogen-associated molecular pattern
PD-1: Programmed death-1
PDL-1: Programmed death ligand-1
PFS: Progression-free survival
PK: Pharmacokinetic
PR: Partial response
PS: Performance status
Q3W: Each 3-week cycle
RECIST: Response Evaluation Criteria in Solid Tumors
SD: Stable disease
t_1/2_: Elimination half life
t_max_: Time of maximum concentration
VEGF: Vascular endothelial growth factor
VEGFR2: Vascular endothelial growth factor receptor 2
Vss: Volume of distribution at steady-state
